# Causal effects of gut microbiota on functional gastrointestinal disorders: a bidirectional Mendelian randomization study

**DOI:** 10.3389/ebm.2026.11023

**Published:** 2026-09-10

**Authors:** Yutong Cheng, Qiuai Shu, Ziwei Wang, Jian Wu, Yuxin Peng, Jiale Xie, Xiru Liang, Zhiwei Tao, Haitao Shi, Zhongcao Wei, Jinhai Wang, Ning Xie

**Affiliations:** 1 Department of Gastroenterology, The Second Affiliated Hospital of Xi’an Jiaotong University, Xi’an, Shaanxi, China; 2 State Key Laboratory of Holistic Integrative Management of Gastrointestinal Cancers, Xijing Hospital of Digestive Diseases, Fourth Military Medical University, Xi’an, Shaanxi, China; 3 Department of Pathology, The Second Affiliated Hospital of Xi’an Jiaotong University, Xi’an, Shaanxi, China; 4 Department of Joint Surgery, HongHui Hospital, Xi’an Jiaotong University, Xi’an, Shaanxi, China; 5 Bioinspired Engineering and Biomechanics Center (BEBC), Xi’an Jiaotong University, Xi’an, Shaanxi, China; 6 The Key Laboratory of Biomedical Information Engineering of Ministry of Education, School of Life Science and Technology, Xi’an Jiaotong University, Xi’an, Shaanxi, China

**Keywords:** causality, functional dyspepsia, functional gastrointestinal disorders, gut microbiota, irritable bowel syndrome

## Abstract

Emerging evidence suggests that gut microbiota is associated with functional gastrointestinal disorders (FGIDs). However, findings regarding microbial alterations in FGIDs have been inconsistent across observational studies, and the direction and causality of these associations remain unclear. We conducted a bidirectional two-sample Mendelian randomization (MR) study to investigate potential causal associations between gut microbiota and two common FGIDs, irritable bowel syndrome (IBS) and functional dyspepsia (FD). Genome-wide association study (GWAS) summary statistics of gut microbiota, IBS, and FD were obtained from public databases and applied to our MR analysis. The inverse-variance-weighted method was used as the primary analysis, with weighted median and complementary sensitivity analyses used to assess the consistency and robustness of the findings. Higher genetically predicted abundances of *order Bifidobacteriales* (OR: 0.741, 95% CI: 0.570 to 0.963, *P* = 0.025) and *genus Eubacterium ventriosum group* (OR: 0.684, 95% CI: 0.524 to 0.893, *P* = 0.005) were associated with a lower risk of IBS. *Genus Lachnospiraceae NK4A136 group* (OR: 1.368, 95% CI: 1.086 to 1.722, *P* = 0.008) correlated to a high risk of FD while *family Desulfovibrionaceae* and *order Desulfovibrionales* (OR: 0.649, 95% CI: 0.471 to 0.893, *P* = 0.008) were protective for FD. In the reverse MR analysis, genetically predicted IBS risk was associated with lower abundances of *Turicibacter* and *Slackia*, whereas no robust reverse associations were observed for FD. These findings identify several microbial taxa as candidates for further mechanistic and clinical validation rather than established risk factors or therapeutic targets. Further experimental research to investigate the underlying mechanisms is warranted.

## Impact statement

This study offers significant insights into the complex causal relationship between gut microbiota and functional gastrointestinal disorders. By utilizing bidirectional Mendelian randomization, we confirm the critical role of gut microbiota in these disorders and identify five microbial taxa that are strongly implicated in their pathogenesis. This work advances the field by providing causal evidence, rather than merely correlational findings, thereby offering a deeper understanding of the underlying mechanisms. The identification of these microbial taxa opens new avenues for therapeutic targeting, potentially leading to innovative strategies for managing and treating functional gastrointestinal disorders. This research not only enhances our understanding of microbiome-host interactions but also paves the way for the development of microbiota-based interventions in gastrointestinal health.

## Introduction

Functional gastrointestinal disorders (FGIDs), also known as disorders of gut–brain interaction (DGBIs), are common symptom-defined gastrointestinal disorders affecting approximately 40% of the global population [1, 2]. Depending on the affected gastrointestinal region, FGIDs may present with symptoms such as abdominal pain, dyspepsia, bloating, diarrhea, constipation, vomiting, and regurgitation. Irritable bowel syndrome (IBS) and functional dyspepsia (FD) are the most common FGIDs, affecting approximately 5%–10% and up to 16% of the general population, respectively [3, 4]. Many FGIDs follow a chronic or relapsing–remitting course and are frequently accompanied by psychological comorbidities, resulting in substantial impairment in quality of life and increased healthcare utilization. However, currently available treatments are largely symptom-directed and often provide only modest or variable benefit [2]. Therefore, a better understanding of the underlying mechanisms of FGIDs is needed to facilitate the development of effective diagnostic and therapeutic strategies.

FGIDs are conceptualized as DGBIs, with complex pathophysiological mechanisms involving gut microbiota, gut motility, the gut mucosal barrier, immune homeostasis, central nervous system, and visceral hypersensitivity [2]. Notably, growing evidence suggests that gut microbiota plays an essential role in maintaining intestinal homeostasis and is influenced by multiple host- and environment-related factors, including diet, age, geography, and health status [5–7]. Alterations in gut microbiota may contribute to FGIDs through immune regulation, microbial metabolites, and gut-brain communication [8–11].

Accordingly, an increasing number of studies have reported alterations in the gut bacterial community in FGIDs, especially in FD and IBS [11–15]. For example, an increase in *Streptococcus* and total bacterial abundance has been found in the duodenum of FD patients [16]. A case-control study reported lower abundances of *Bacteroides* and *Parabacteroides sp.* in patients with IBS than in healthy controls [17]. In contrast, a probiotic intervention study found a higher baseline abundance of *Bacteroides* in patients with IBS than in healthy controls, which decreased after probiotic treatment [18]. These inconsistent findings underscore the substantial heterogeneity of observational microbiome studies, and it remains unclear whether the reported microbial alterations are causes, consequences, or correlates of FGIDs [2, 19, 20].

Mendelian randomization (MR) adopts genetic variants as instrumental variables (IVs) to assess whether an observational association between an exposure and an outcome is consistent with a causal effect [21]. As genetic variants are randomly allocated at meiosis and always stable, MR can minimize the confounding. In this study, we use single nucleotide polymorphisms (SNPs) from the existing genome-wide association study (GWAS) databank to perform a bidirectional MR study for determining the causal associations between gut microbiota and FGIDs.

## Materials and methods

### Study design

**FIGURE 1:**
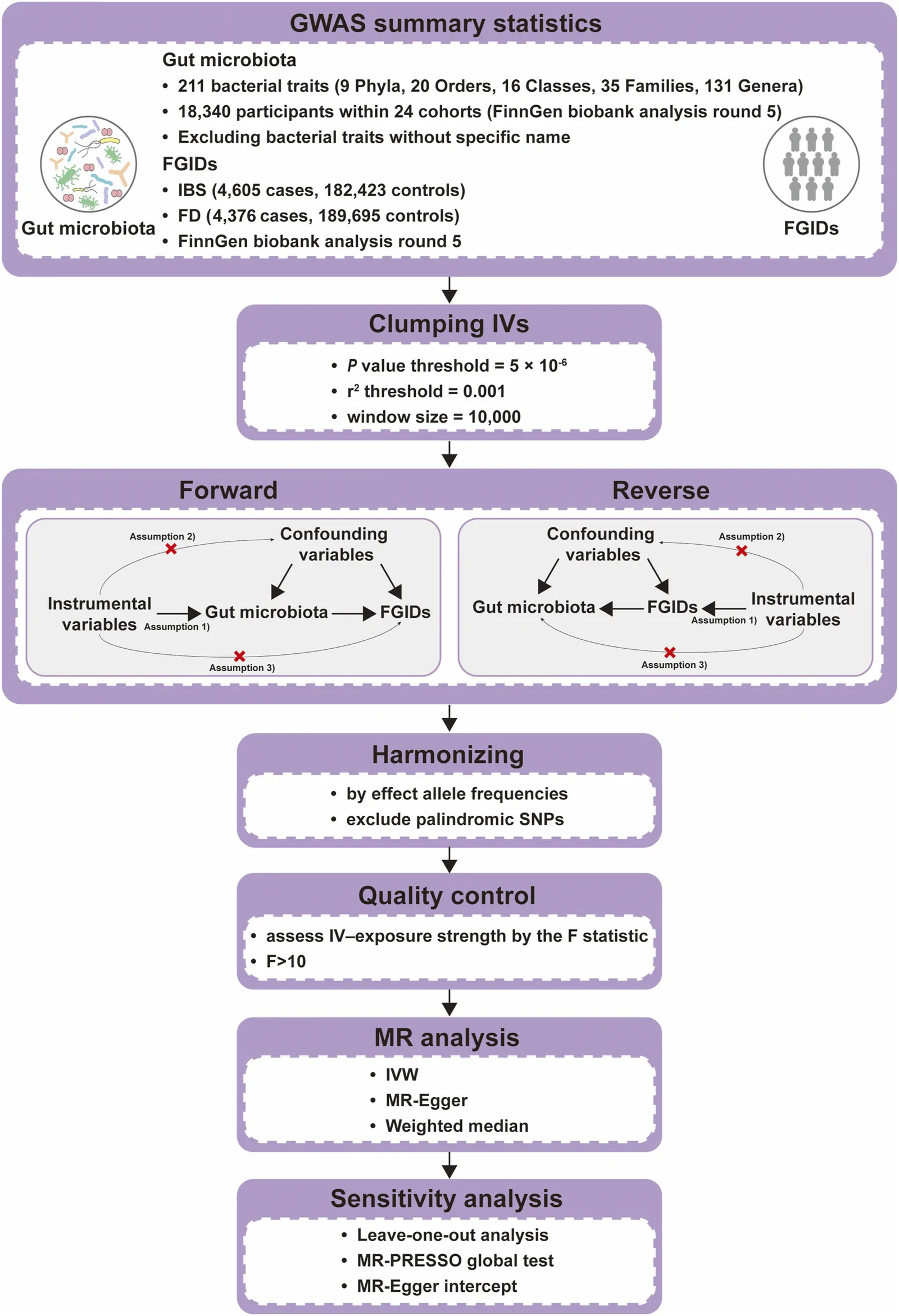
Workflow of our two-sample bidirectional MR analysis. GWAS, Genome-wide association study; FGIDs, Functional gastrointestinal disorders; IBS, Irritable bowel syndrome; FD, Functional dyspepsia; IVW, Inverse-variance-weighted; MR-PRESSO, Mendelian Randomization Pleiotropy RESidual Sum and Outlier.

We employed a bidirectional two-sample Mendelian randomization analysis, which used the genetic variants as the instrumental variables to explore the potential causal relationships between gut microbiota and FGIDs. The validity of MR analysis relies on three core assumptions [22]. First, the selected IVs should be strongly associated with the exposure. This assumption was addressed by selecting SNPs based on predefined significance thresholds, performing linkage disequilibrium (LD) clumping, and evaluating instrument strength using the F statistic. Second, the IVs should not be associated with potential confounding factors. This assumption was further evaluated by screening IVs involved in significant MR associations against potential confounding traits in the GWAS Catalog. Third, the IVs should influence the outcome only through the exposure. This assumption was assessed using multiple sensitivity analyses, including MR-Egger regression, MR-PRESSO, Cochran’s Q test, and leave-one-out analysis. A flowchart exhibits the study design in Figure 1.

### Data sources

GWAS statistics with a sample size of 14,306 cases of gut microbiota were obtained from the OpenGWAS database (https://gwas.mrcieu.ac.uk/). Since 15 bacterial traits did not have their specific species names, we excluded them and selected 196 bacterial traits classified into specific phylum, class, order, family, and genus.

The GWAS summary statistics for FGIDs (*i.e.*, IBS and FD) were obtained from the FinnGen biobank accessed through the OpenGWAS database (https://gwas.mrcieu.ac.uk/). Both phenotypes were defined according to the FinnGen registry-based endpoint definitions, which are based on the International Classification of Diseases (ICD) coding system. Specifically, IBS was defined using ICD-10 code K58, ICD-9 code 5641, and ICD-8 code 56419, while FD was defined using ICD-10 code K30, ICD-9 code 5368A, and ICD-8 code 5361. The summarized data for IBS was derived from 187,028 individuals including 4,605 cases and 182,423 controls, covering 16,380,380 SNPs, whereas those for FD were derived from 194,071 individuals comprising 4,376 cases and 189,695 controls, covering 16,380,376 SNPs. All participants were of European ancestry. The GWAS datasets for gut microbiota and FGIDs were derived from independent research consortia, making substantial sample overlap unlikely. However, as individual-level participant information was unavailable, complete verification of participant overlap was not possible. There was no additional requirement for ethical approval or informed consent, as the data used in this study was all from public available summary-level GWAS data.

### IVs selection

For IVs, we adopted several quality control criteria to select applicable genetic variants. First, we selected candidate SNPs at the genome-wide significance level of *P* < 5.0 × 10^−8^. However, the number of eligible SNPs was too small. We employed a relatively more relaxed threshold (*P* < 5.0 × 10^−6^) to obtain a more accurate result. Next, to reduce linkage disequilibrium (LD), we set a threshold of r^2^ < 0.001 and a window size of 10,000 kb for clumping [23]. Finally, we harmonized the respective exposure and outcome datasets and excluded palindromic SNPs with intermediate allele frequencies. To further evaluate the independence assumption, IVs involved in significant MR associations were additionally screened in the GWAS catalog for genome-wide significant associations with potential confounding traits, including body mass index, diet-related traits, smoking, medication use, psychiatric conditions, and other gastrointestinal diseases.

We calculated the F-statistic of IVs to ensure their strength for exposure. An F-statistic >10 could minimize the weak instrument bias in the model. F-statistics were calculated as F = R^2^ × [(N-1-k)/k]/(1-R^2^), in which R^2^ and N denoted the accumulated variance explained by specific SNPs and sample size, respectively [24]. We calculated R^2^ according to the following formula [25]:

$$R^{2}=(2\times \text{EAF}\times \left(1-\text{EAF}\right)\times \beta^{2}/[(2\times \text{EAF}\times \left(1-\text{EAF}\right)\times \beta^{2}+\left(2\times \text{EAF}\times \left(1-\text{EAF}\right)\times N\times {\text{SE}}^{2}\right)$$

### MR analysis and statistical analysis

To investigate the potential causal relationship between gut microbiota and FGIDs, we adopted several approaches to perform the bidirectional two-sample MR analysis in our study. At first, the inverse-variance-weighted (IVW) method was performed as the primary analysis to measure the causal relationship between exposure and outcome. We subsequently used weighted median as an auxiliary method. MR-Egger regression was performed to evaluate the horizontal pleiotropy by intercept test.

Several sensitivity analyses were conducted to evaluate the robustness of the MR results and the validity of the exclusion restriction assumption. We employed Cochran’s Q test to evaluate the heterogeneity among SNPs involved in each analysis. We performed the leave-one-out sensitivity analysis to measure the robustness of our results. MR-Egger was used to evaluate the potential pleiotropy in the IVW model, and MR-PRESSO was performed to test and correct the horizontal pleiotropy by removing outlying instrumental variables (NbDistribution = 3,000, SignifThreshold = 0.05). All the estimates as odds ratio (OR) were shown with their 95% confidence intervals (CIs) per one standard deviation (SD) increase in the exposures.

We conducted all statistical analyses above in R 4.1.3 with the R package of “TwoSampleMR” (version 0.5.6) and “MRPRESSO”. *P* < 0.05 was considered statistically significant evidence for a potential causal effect. This study was reported in accordance with the Strengthening the Reporting of Observational Studies in Epidemiology Using Mendelian Randomization (STROBE-MR) guideline, and the completed checklist is provided in Supplementary Material 1.

## Results

### IVs

In the analysis exploring the role of gut microbiota in FGIDs, 1-12 SNPs were selected for gut microbiota species as IVs. After clumping linkage disequilibrium and removing palindromic SNPs, six SNPs and two SNPs were selected as IVs of IBS and FD to identify the effect of FGIDs on gut microbiota, respectively. As there were no SNPs left after harmonization, some analysis failed. The F statistics of IVs indicated that there was no bias on account of weak instruments (F > 10, Supplementary Tables S1, S2). For the significant MR associations, no IVs were identified as requiring exclusion after screening for potential confounding traits (Supplementary Table S3).

### MR analysis of FGIDs and gut microbiota

**FIGURE 2:**
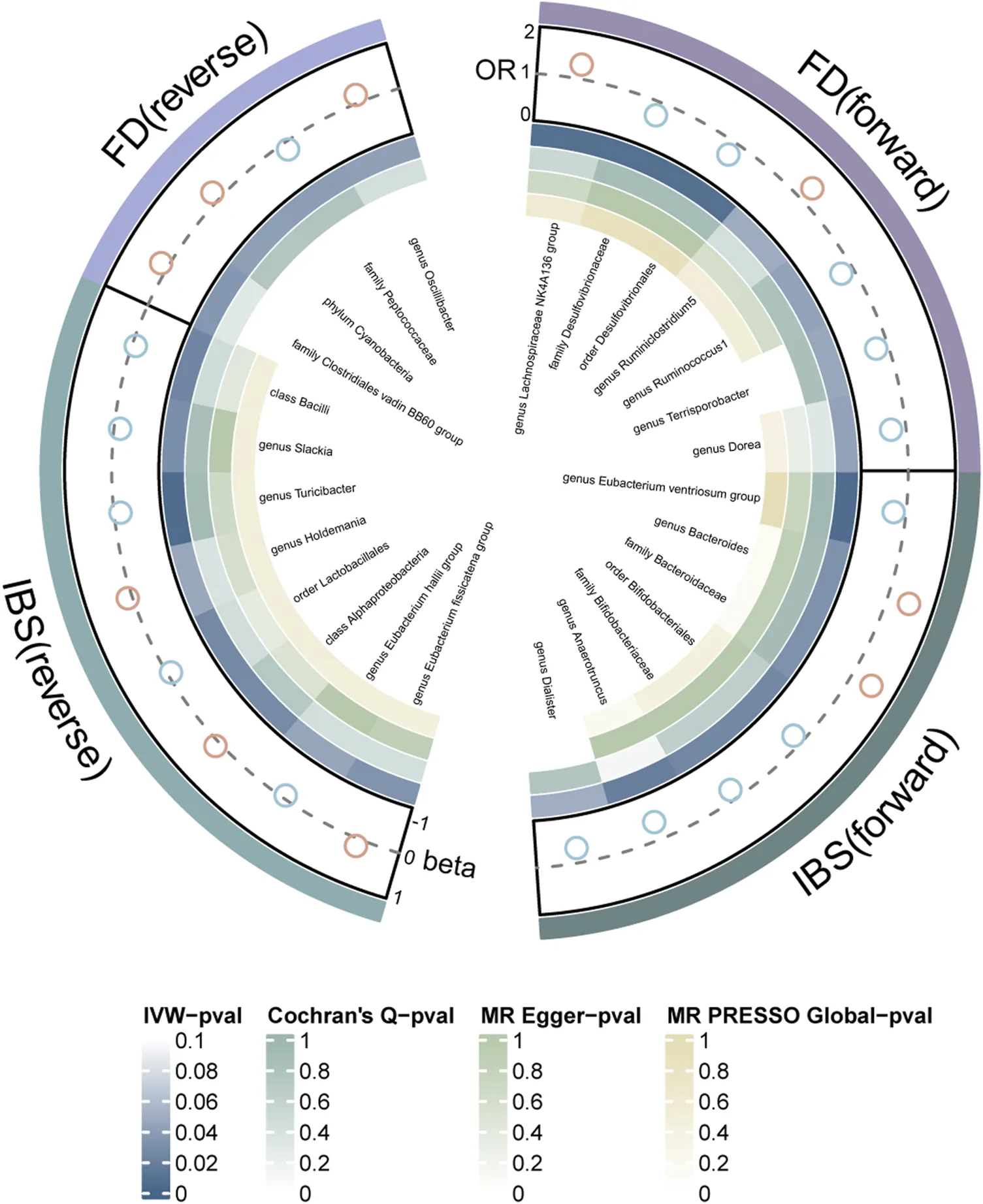
All results of MR analysis and sensitivity analysis between identified gut microbiota and FGIDs (Genome-wide statistical significance, *P* < 5 × 10^−6^).

**FIGURE 3:**
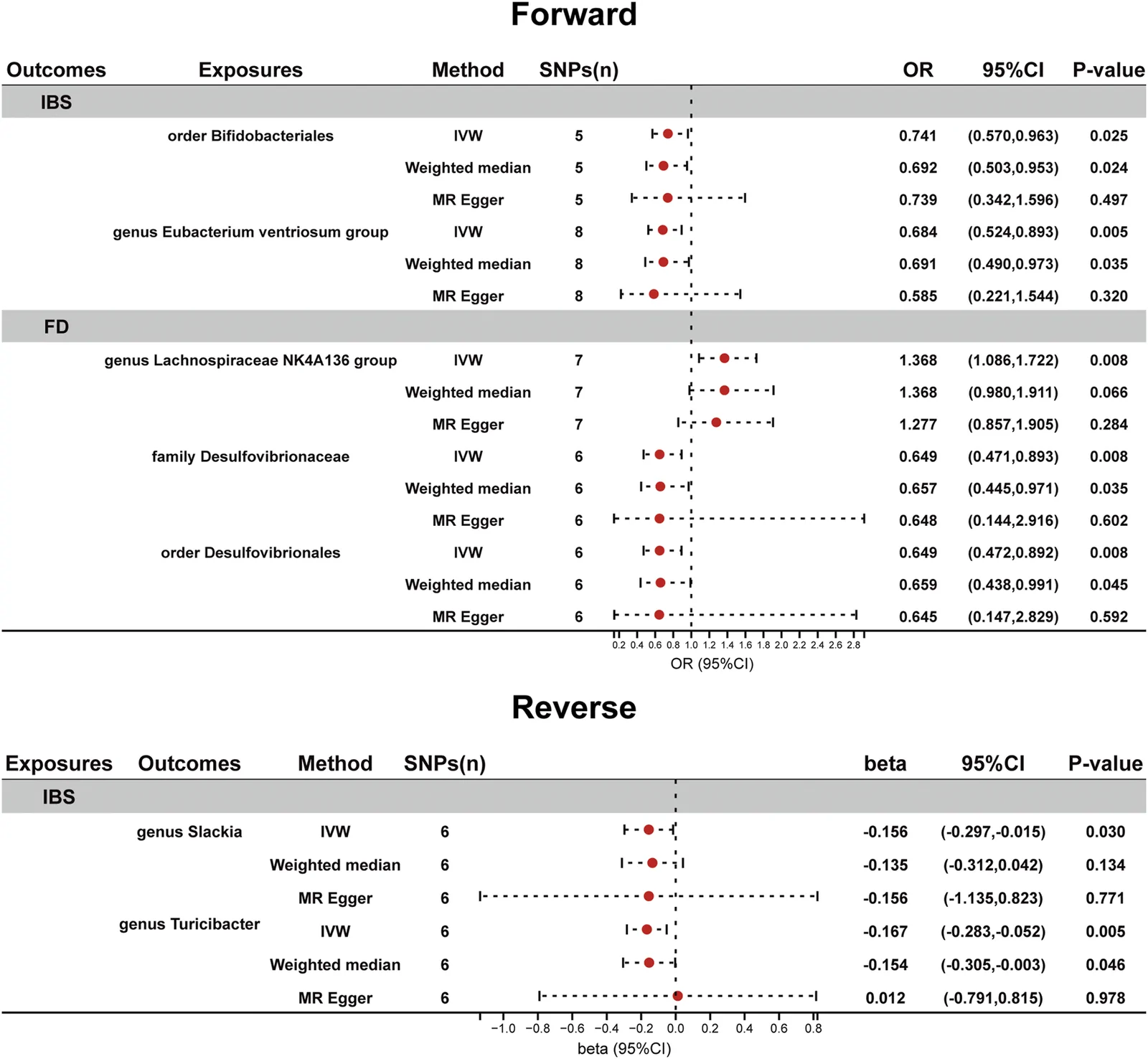
Forest plot of the causal relationship between seven microbial taxa and FGIDs (IBS and FD) in a bidirectional MR analysis. OR: odds ratio; 95% CI: 95% confidence interval.

**FIGURE 4:**
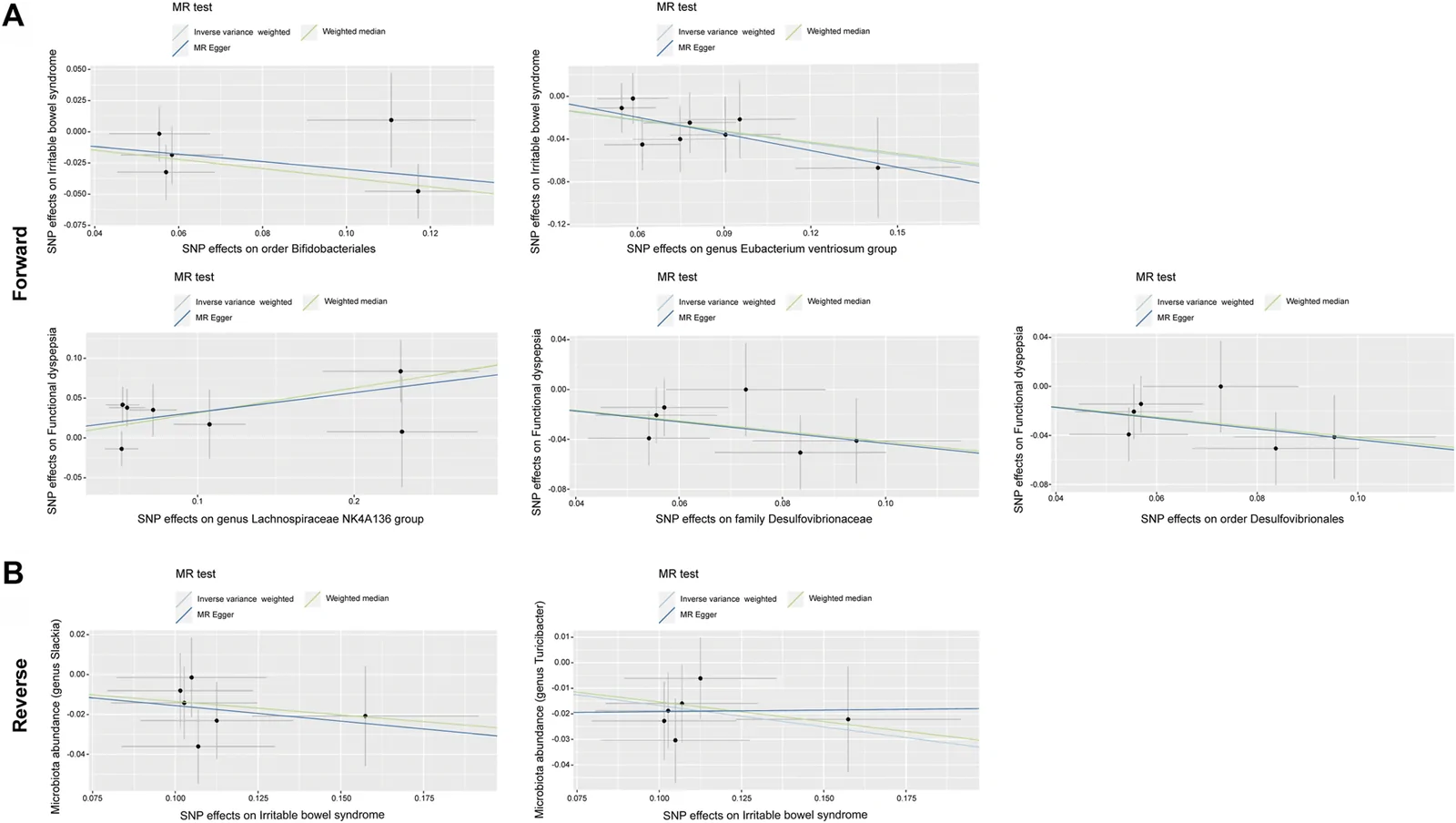
Scatter diagram of the causal relationship between seven genetically identified microbial taxa and FGIDs (IBS and FD) in a bidirectional MR analysis. **(A)** Causal effects of gut microbiota on IBS and FD; **(B)** Causal effects of IBS on gut microbiota. The estimations (95% CI) were coefficients and corresponding to a 95% confidence interval.

Based on two-sample MR analysis, the IVW analysis identified associations between the genetically predicted abundances of 14 microbial taxa and the risk of FGIDs; only five microbial taxa remained robust in sensitivity analyses (Figures 2–4; Table 1). The complete results are shown in Supplementary Tables S1, S4. To clarify the hierarchy of evidence, we further classified these associations according to whether they were supported by both the IVW and weighted median methods. For IBS, the protective associations of *order Bifidobacteriales* and *genus Eubacterium ventriosum group* were supported by both estimators. For *order Bifidobacteriales*, the IVW estimate indicated a lower risk of IBS (OR: 0.741, 95% CI: 0.570 to 0.963, *P* = 0.025), with a consistent and statistically significant weighted median estimate (OR: 0.692, 95% CI: 0.503 to 0.953, *P* = 0.024). Similarly, *genus Eubacterium ventriosum group* was inversely associated with IBS in both the IVW analysis (OR: 0.684, 95% CI: 0.524 to 0.893, *P* = 0.005) and the weighted median analysis (OR: 0.691, 95% CI: 0.490 to 0.973, *P* = 0.035). For FD, the protective associations of *family Desulfovibrionaceae* and *order Desulfovibrionales* were supported by both estimators. *Family Desulfovibrionaceae* was inversely associated with the risk of FD in both the IVW analysis (OR: 0.649, 95% CI: 0.471 to 0.893, *P* = 0.008) and the weighted median analysis (OR: 0.657, 95% CI: 0.445 to 0.971, *P* = 0.035). A similar protective association was observed for *order Desulfovibrionales* using the IVW method (OR: 0.649, 95% CI: 0.472 to 0.892, *P* = 0.008) and the weighted median method (OR: 0.659, 95% CI: 0.438 to 0.991, *P* = 0.045). A higher abundance of *genus Lachnospiraceae NK4A136 group* was associated with an increased risk of FD in the IVW analysis (OR: 1.368, 95% CI: 1.086 to 1.722, *P* = 0.008). Although the weighted median estimate was directionally consistent, it did not reach statistical significance (OR: 1.368, 95% CI: 0.980 to 1.911, *P* = 0.066).

**TABLE 1:** All results of the sensitivity analyses between seven identified microbial taxa and FGIDs.

Exposure	Outcomes	MR-PRESSO	IVW estimates		MR-egger pleiotropy test	
		Global *P*-value	Cochran’s Q	*P*-value	MR-egger intercept	*P*-value
Order bifidobacteriales	IBS	0.440	2.528	0.640	<0.001	0.993
Genus eubacterium ventriosum group	IBS	0.979	2.113	0.953	0.012	0.752
Genus lachnospiraceae NK4A136 group	FD	0.609	5.093	0.532	0.008	0.698
Family Desulfovibrionaceae	FD	0.887	1.681	0.891	<0.001	1.000
Order Desulfovibrionale	FD	0.906	1.661	0.894	<0.001	0.993
IBS	Genus Slackia	0.492	1.930	0.859	<0.001	1.000
IBS	Genus Turicibacter	0.498	1.442	0.920	−0.020	0.681

Then, we calculated Cochran’s Q statistics, which showed there was little evidence for heterogeneity among the SNP-specific causal estimates for the identified associations, as shown in Supplementary Figure S1A. Moreover, the MR-Egger intercept tests provided no evidence of directional horizontal pleiotropy (intercept *P* = 0.993 for *order Bifidobacteriales*, intercept *P* = 0.752 for *genus Eubacterium ventriosum group*, intercept *P* = 0.993 for *genus Lachnospiraceae NK4A136 group*, intercept *P* = 1.000 for *family Desulfovibrionaceae*, as well as intercept *P* = 0.698 for *order Desulfovibrionales*). In addition, the MR-PRESSO global tests detected no evidence of overall horizontal pleiotropy, and no outlier SNPs were identified. Leave-one-out analyses indicated that none of the associations were disproportionately driven by any single SNP.

### Reverse MR analysis of FGIDs and gut microbiota

In our reverse MR analysis, the IVW method initially identified associations of genetically predicted FGIDs risk with the abundance of 12 microbial taxa, as shown in Figure 2. Only two associations involving IBS remained robust after sensitivity analyses, whereas none of the IVW-positive associations involving FD remained robust. Thus, robust reverse MR associations were observed only for IBS. Comprehensive results were shown in Supplementary Tables S1, S4. Genetically predicted IBS risk was associated with a lower abundance of *genus Turicibacter* using both the IVW method (Beta: −0.167, 95% CI: −0.283 to −0.052, *P* = 0.005) and the weighted median method (Beta: −0.154, 95% CI: −0.305 to −0.003, *P* = 0.046). Genetically predicted IBS risk was also associated with a lower abundance of *genus Slackia* in the IVW analysis (Beta: −0.156, 95% CI: −0.297 to −0.015, *P* = 0.030). Although the weighted median estimate was directionally consistent, it was not statistically significant (Beta: −0.135, 95% CI: −0.312 to 0.042, *P* = 0.134). As shown in Supplementary Figure S1B, neither between-SNP-heterogeneity nor directional pleiotropy could be identified in Cochran’s Q test and MR-Egger analysis. Leave-one-out analyses indicated that neither association was disproportionately influenced by a single genetic variant.

## Discussion

**FIGURE 5:**
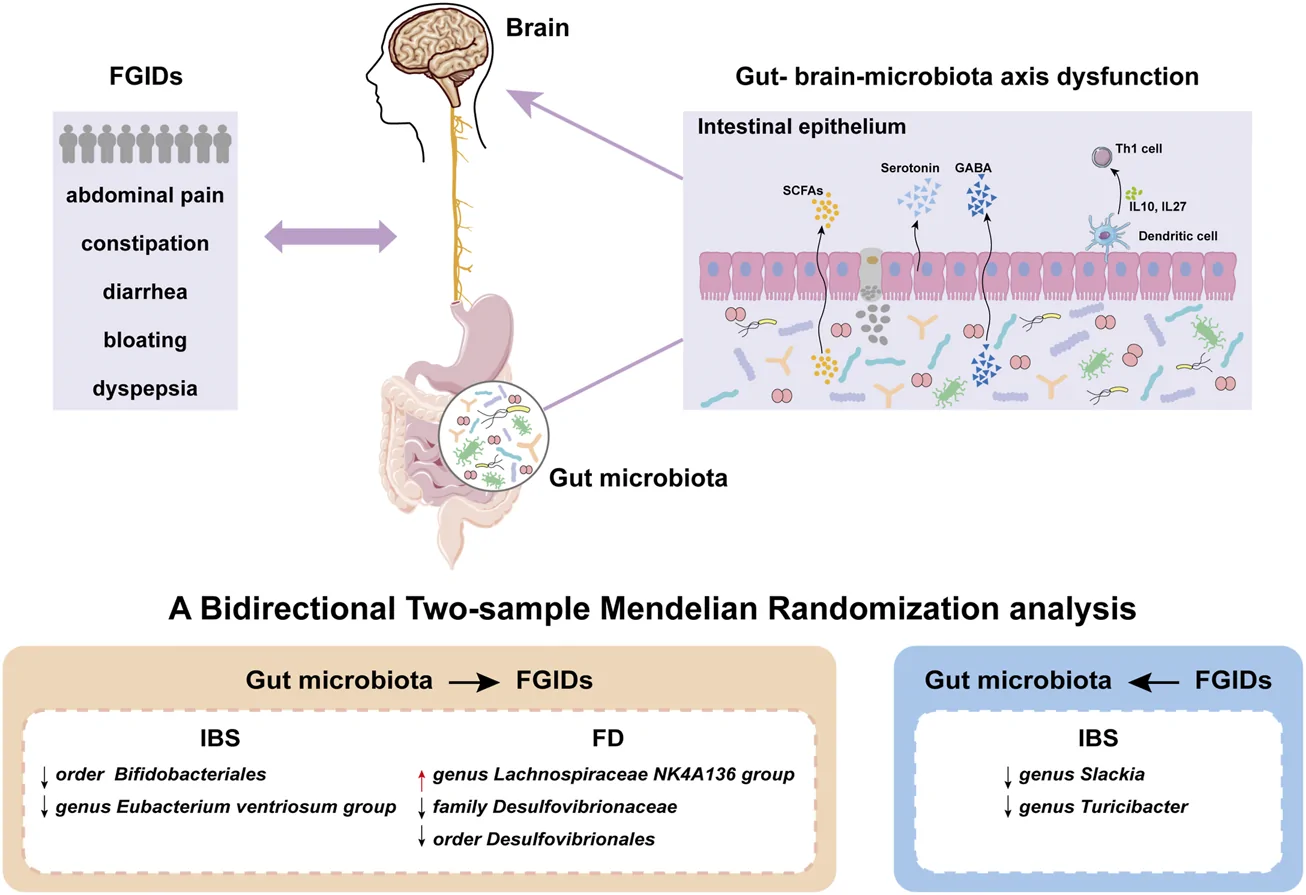
Schematic representation of the present study.

In the current study, we conducted a bidirectional two-sample MR study to investigate the potential causal relationships and directions of association between gut microbiota and FGIDs, particularly IBS and FD (Figure 5). First, we identified that *genus Eubacterium ventriosum group* and *order Bifidobacteriales* were negatively associated with IBS. Second, we found that a higher genetically predicted abundance of *genus Lachnospiraceae NK4A136 group* was associated with an increased risk of FD, whereas higher genetically predicted abundances of *family Desulfovibrionaceae* and *order Desulfovibrionales* were associated with a lower risk of FD. Finally, reverse MR analysis showed that genetically predicted IBS risk was associated with lower abundances of *genus Turicibacter* and *genus Slackia.*

Accumulating evidence suggests that gut microbial dysbiosis is involved in the pathophysiology of gastrointestinal disorders, including IBS and FD. A systematic review reported a lower abundance of the *genus Bifidobacterium* in patients with IBS than in healthy controls, although the findings were not uniform across studies [19]. This observational pattern is directionally consistent with our MR finding that a higher genetically predicted abundance of the *order Bifidobacteriales* was associated with a lower risk of IBS, although the taxonomic levels are not directly equivalent. As reported, several mechanisms may provide biological plausibility for the inverse association between specific members of *Bifidobacteriales* and IBS. Certain *Bifidobacterium* species or strains can generate γ-amino butyric acid (GABA), an inhibitory neurotransmitter [26, 27], and this activity may influence visceral sensitivity through the microbiota–gut–brain axis, thereby providing a possible biological mechanism relevant to abdominal pain [28]. Consistent with a potential beneficial role of selected *Bifidobacterium* strains, a randomized controlled trial reported that *Bifidobacterium,* at a specific dose, improved abdominal pain or discomfort in women with IBS [29]. In addition, several randomized trials of *Bifidobacterium*-containing fermented milk products or multispecies probiotic formulations have reported improvements in bowel habits, abdominal distension, or global IBS symptoms [30–33]. However, these effects cannot necessarily be attributed specifically to the *order Bifidobacteriales*. Moreover, abnormal activation of the immune system has been identified as a characteristic in a subset of IBS patients [34]. In an experimental study, *Bifidobacterium breve* activated intestinal CD103+ dendritic cells through a TLR2/MyD88-dependent pathway, promoting IL-10- and IL-27-mediated differentiation of IL-10-producing type 1 regulatory T (Tr1) cells [35].

In addition, our MR analysis showed that a higher genetically predicted abundance of the *genus Eubacterium ventriosum group* was associated with a lower risk of IBS. The *Eubacterium ventriosum group* was also decreased in inflammatory bowel disease (IBD) and showed an inverse association with IBD in a previous two-sample MR analysis [36, 37]. Although these findings suggest a potential role in intestinal health, evidence from IBD provides only indirect support for its relevance to IBS. A butyrate-producing human fecal isolate was found to be phylogenetically closely related to *E. ventriosum* [38]. Butyrate supports epithelial barrier integrity and mucosal immune regulation and may modulate intestinal motility and visceral sensitivity, although these effects are context-dependent [39–42]. These properties provide an indirect biological rationale for the observed association. However, *Eubacterium* is phylogenetically, phenotypically, and metabolically heterogeneous [43], and the function of a single related isolate cannot be generalized to the GWAS-defined *Eubacterium ventriosum group*. Direct clinical evidence linking this taxon to IBS symptoms or treatment response remains limited. This association should therefore be regarded as hypothesis-generating and warrants strain-resolved functional studies and direct clinical validation.

Our reverse MR analysis suggested that genetically predicted IBS risk was associated with lower abundances of *genus Turicibacter* and *genus Slackia*. Consistent with the direction of the MR estimate, a longitudinal observational study reported a lower abundance of *Turicibacter* in patients with IBS than in controls [44]. Experimental evidence that *Turicibacter* interacts bidirectionally with host serotonergic signaling provides a possible mechanistic link to the microbiota–gut–brain axis [45].

Accumulating evidence also implicates alterations in the gut microbial community in FD [4, 15]. Our MR study showed that a higher genetically predicted abundance of *genus Lachnospiraceae NK4A136 group* was associated with an increased risk of FD. Similarly, Zhang et al. reported an increased abundance of the *Lachnospiraceae NK4A136 group* in mice with dyspepsia [46], providing limited preclinical support for the direction of our finding. However, this association has not been confirmed in patients with FD. Several members of the broader *Lachnospiraceae* family are involved in short-chain fatty acid metabolism, and SCFAs can modulate epithelial barrier function and mucosal immune responses [47, 48]. Nevertheless, the metabolic capacity of the GWAS-defined *Lachnospiraceae NK4A136 group* remains poorly characterized, and these general family-level properties do not explain why a higher genetically predicted abundance of this taxon was associated with an increased risk of FD. Previous studies have reported potentially protective effects of *Lachnospiraceae NK4A136 group* and SCFAs on ulcerative colitis and arthritis in mice [49, 50]. These contrasting findings may reflect differences in disease context and anatomical site.

Higher genetically predicted abundances of the *family Desulfovibrionaceae* and the *order Desulfovibrionales* were associated with a lower risk of FD. *Desulfovibrionaceae* belongs to *Desulfovibrionales*, and both analyses used the same seven instrumental SNPs and yielded nearly identical estimates. The findings are therefore likely to represent highly overlapping rather than independent signals. Available human gastric and duodenal microbiome studies have not provided direct evidence linking either taxon to FD [51]. However, in an FD rat model, *fecal Desulfovibrio* abundance was increased after a multicomponent intervention that also improved dyspepsia-related measures [52]. This provides limited, directionally consistent preclinical evidence but does not establish an independent effect of this genus.

FD primarily involves the gastroduodenal region [4], whereas the microbiome GWAS used in our analysis was based predominantly on fecal samples, which may not represent the microbial composition or activity at the relevant disease site. These FD-related associations should therefore be considered exploratory and require validation using anatomically relevant sampling and strain-resolved functional analyses.

In the past decades, a series of clinical trials evaluating the effects of antibiotics (mostly rifaximin and neomycin), prebiotics, probiotics, FMT, and dietary treatments on FGIDs yielded unsatisfactory results, as the improvement of these therapies on gastrointestinal symptoms was limited [53–55]. Up to now, treatments for FGIDs have mainly been symptom-relieving therapies [4]. Our study provides genetic evidence supporting potential causal associations between specific gut microbial taxa and IBS or FD, thereby improving our understanding of the contribution of gut microbial dysbiosis to these disorders. Although the present MR findings do not demonstrate that direct supplementation, depletion, or therapeutic modulation of these taxa would improve FGID symptoms, they may help prioritize candidate taxa for subsequent functional characterization and clinical validation. Future studies should replicate these associations in independent and ancestrally diverse cohorts. Strain-resolved shotgun metagenomics, anatomically relevant sampling, and integrated metatranscriptomic and metabolomic analyses are needed to identify the microbial strains, functional pathways, and metabolites underlying these associations. Candidate strains and metabolites should then be evaluated in complementary mechanistic models, including intestinal organoids and gnotobiotic or humanized animal models, before being advanced to adequately powered, well-controlled intervention trials assessing safety, target engagement, and clinically meaningful outcomes in well-phenotyped patients with IBS or FD. Such stepwise validation could ultimately inform the development of more targeted and individualized microbiota-based strategies for FGIDs.

A key strength of this study is the use of a bidirectional two-sample MR design, together with complementary sensitivity analyses, to evaluate the potential causal relationships between gut microbiota and IBS or FD. Inevitably, there are some drawbacks to our study. First, due to the lack of the GWASs data on other FGIDs subtypes, we only analyzed the causal relationship between gut microbiota and IBS/FD. Similarly, the incomplete data prevented us from conducting IBS subtype-specific and FD subtype-specific analyses. Second, the number of SNPs as IVs for gut microbiota was limited, which may lead to weak-instrument bias. Third, we can only analyze the microbial taxa at the level from genus to phylum constrained by the 16S rRNA gene sequencing results and may miss some specific bacteria that could play a crucial role in the pathogenesis of FGIDs. Furthermore, genetic instruments may not reflect dynamic changes in gut microbiota across different disease stages, environmental exposures, or treatment periods. Fourth, MR evidence alone cannot determine whether modulation of the identified microbial taxa would confer clinical benefits; this possibility requires validation in mechanistic studies and well-designed clinical trials. Fifth, because the GWAS datasets were derived predominantly from populations of European ancestry, the generalizability of our findings to Asian and other ancestry groups remains uncertain. Future studies using more ancestrally diverse GWAS datasets are warranted.

## Conclusion

In summary, our study identified potential bidirectional associations between gut microbiota and FGIDs, with robust reverse associations observed mainly for IBS, and highlighted several microbial taxa as candidates for future mechanistic and clinical validation rather than established therapeutic targets. Further studies are required to clarify the complex interactions between gut microbiota and FGIDs and to determine their potential relevance for disease mechanisms and microbiota-based interventions.

## Data Availability

The original contributions presented in the study are included in the article/Supplementary Material, further inquiries can be directed to the corresponding authors.
